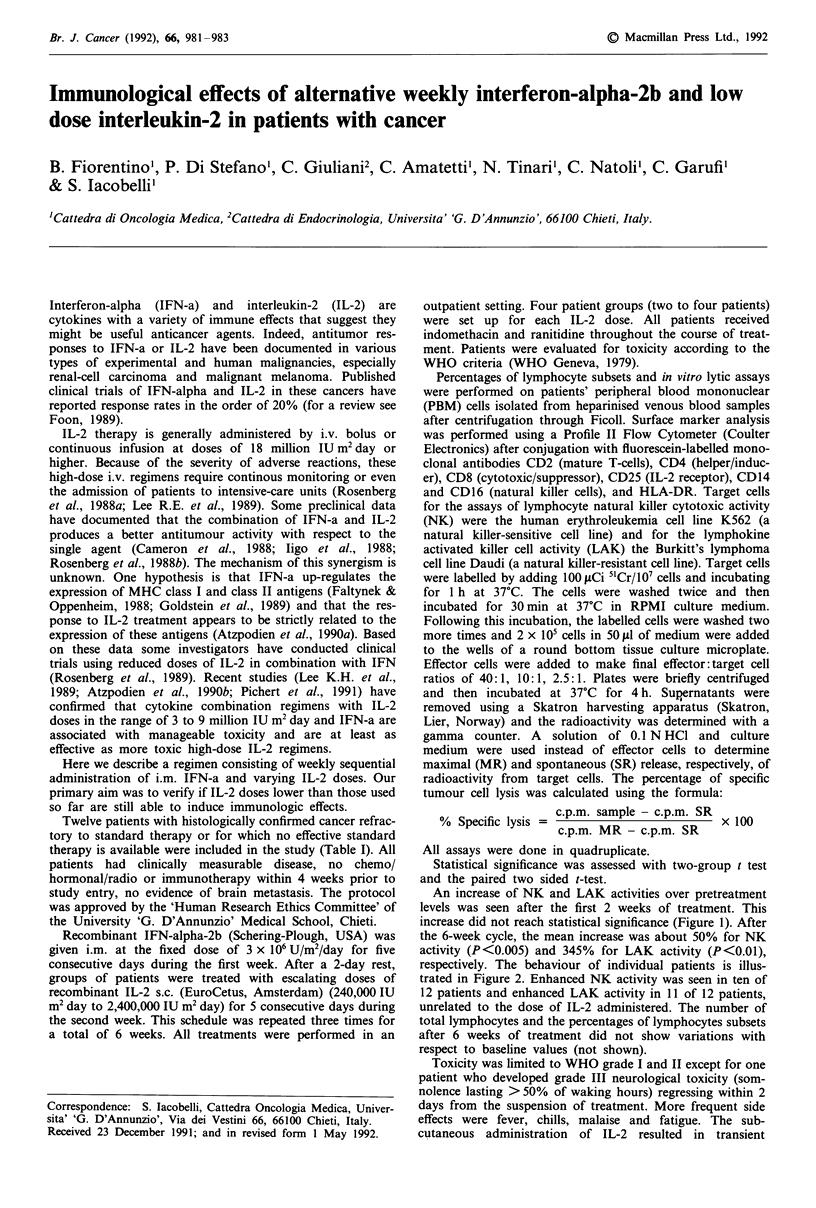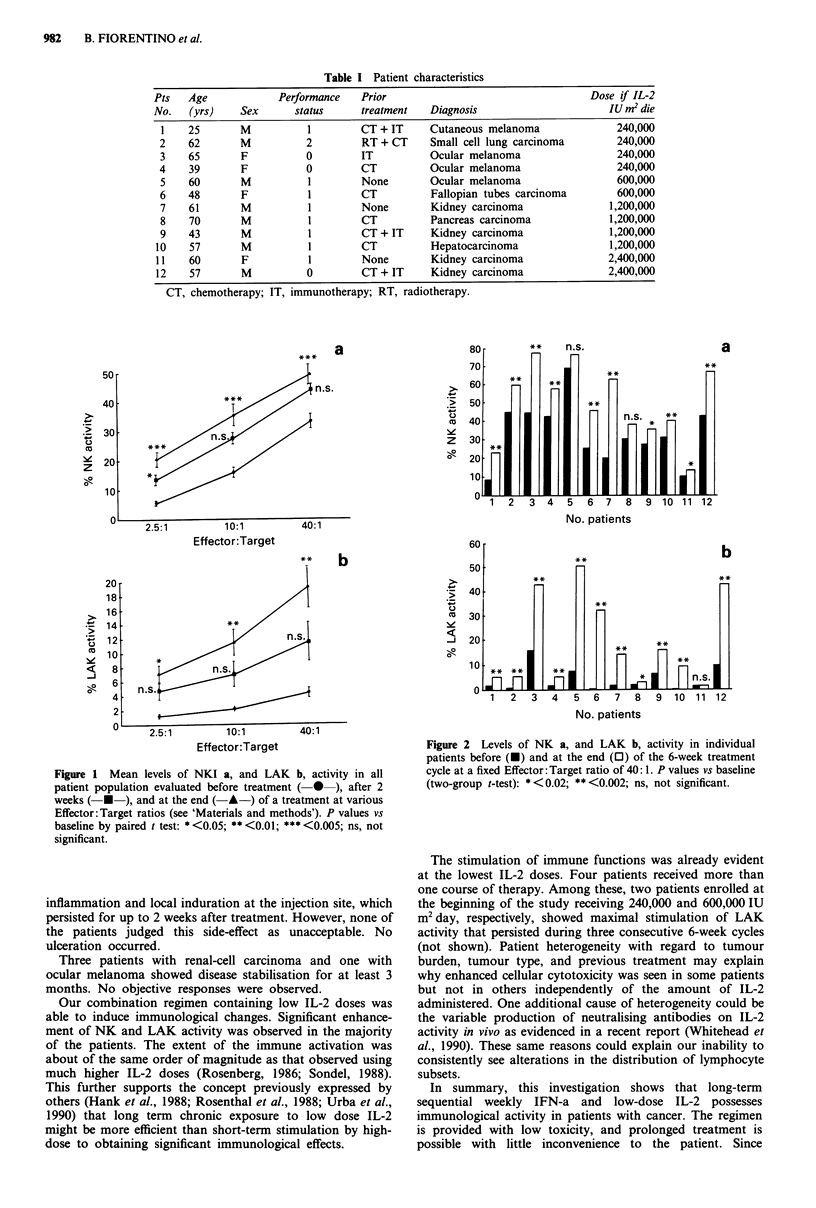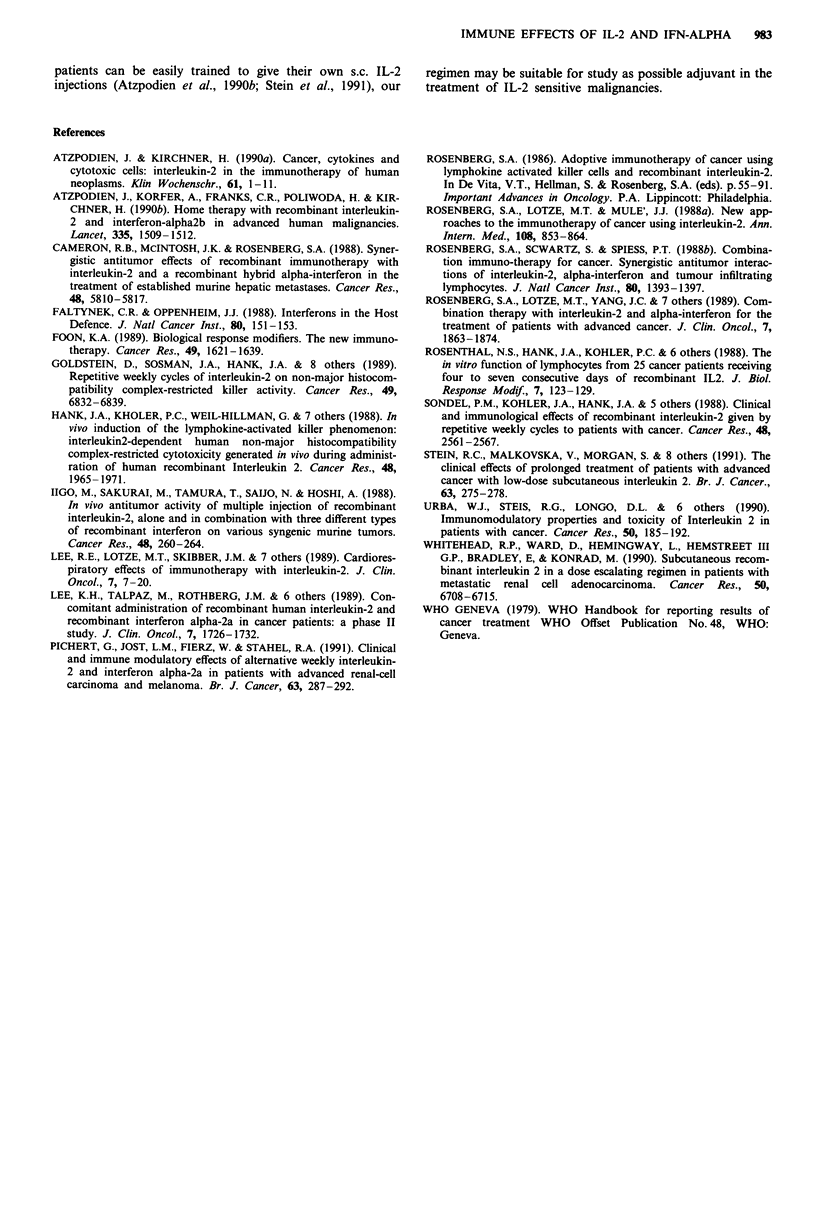# Immunological effects of alternative weekly interferon-alpha-2b and low dose interleukin-2 in patients with cancer.

**DOI:** 10.1038/bjc.1992.396

**Published:** 1992-11

**Authors:** B. Fiorentino, P. Di Stefano, C. Giuliani, C. Amatetti, N. Tinari, C. Natoli, C. Garufi, S. Iacobelli

**Affiliations:** Cattedra di Oncologia Medica, Chieti, Italy.


Br. J. Cancer (1992), 66, 981-983 © Macmillan Press Ltd., 1992

### Immunological effects of alternative weekly interferon-alpha-2b and low dose interleukin-2 in patients with cancer

B. Fiorentino[1], P. Di Stefano[1], C. Giuliani[2], C. Amatetti[1], N. Tinari[1], C. Natoli[1], C. Garufi[1] & S. Iacobelli[1]

[1]*Cattedra di Oncologia Medica,* [2]*Cattedra di Endocrinologia, Universita' 'G. D'Annunzio', 66100 Chieti, Italy.*

Interferon-alpha (IFN-a) and interleukin-2 (IL-2) are cytokines with a variety of immune effects that suggest they might be useful anticancer agents. Indeed, antitumor responses to IFN-a or IL-2 have been documented in various types of experimental and human malignancies, especially renal-cell carcinoma and malignant melanoma. Published clinical trials of IFN-alpha and IL-2 in these cancers have reported response rates in the order of 20% (for a review see Foon, 1989).

IL-2 therapy is generally administered by i.v. bolus or continuous infusion at doses of 18 million IU $m^2$ day or higher. Because of the severity of adverse reactions, these high-dose i.v. regimens require continous monitoring or even the admission of patients to intensive-care units (Rosenberg *et al.*, 1988*a*; Lee R.E. *et al.*, 1989). Some preclinical data have documented that the combination of IFN-a and IL-2 produces a better antitumour activity with respect to the single agent (Cameron *et al.*, 1988; Iigo *et al.*, 1988; Rosenberg *et al.*, 1988*b*). The mechanism of this synergism is unknown. One hypothesis is that IFN-a up-regulates the expression of MHC class I and class II antigens (Faltynek & Oppenheim, 1988; Goldstein *et al.*, 1989) and that the response to IL-2 treatment appears to be strictly related to the expression of these antigens (Atzpodien *et al.*, 1990*a*). Based on these data some investigators have conducted clinical trials using reduced doses of IL-2 in combination with IFN (Rosenberg *et al.*, 1989). Recent studies (Lee K.H. *et al.*, 1989; Atzpodien *et al.*, 1990*b*; Pichert *et al.*, 1991) have confirmed that cytokine combination regimens with IL-2 doses in the range of 3 to 9 million IU $m^2$ day and IFN-a are associated with manageable toxicity and are at least as effective as more toxic high-dose IL-2 regimens.

Here we describe a regimen consisting of weekly sequential administration of i.m. IFN-a and varying IL-2 doses. Our primary aim was to verify if IL-2 doses lower than those used so far are still able to induce immunologic effects.

Twelve patients with histologically confirmed cancer refractory to standard therapy or for which no effective standard therapy is available were included in the study (Table I). All patients had clinically measurable disease, no chemo/hormonal/radio or immunotherapy within 4 weeks prior to study entry, no evidence of brain metastasis. The protocol was approved by the 'Human Research Ethics Committee' of the University 'G. D'Annunzio' Medical School, Chieti.

Recombinant IFN-alpha-2b (Schering-Plough, USA) was given i.m. at the fixed dose of $3 \times 10^6$ U/$m^2$/day for five consecutive days during the first week. After a 2-day rest, groups of patients were treated with escalating doses of recombinant IL-2 s.c. (EuroCetus, Amsterdam) (240,000 IU $m^2$ day to 2,400,000 IU $m^2$ day) for 5 consecutive days during the second week. This schedule was repeated three times for a total of 6 weeks. All treatments were performed in an outpatient setting. Four patient groups (two to four patients) were set up for each IL-2 dose. All patients received indomethacin and ranitidine throughout the course of treatment. Patients were evaluated for toxicity according to the WHO criteria (WHO Geneva, 1979).

Percentages of lymphocyte subsets and *in vitro* lytic assays were performed on patients' peripheral blood mononuclear (PBM) cells isolated from heparinised venous blood samples after centrifugation through Ficoll. Surface marker analysis was performed using a Profile II Flow Cytometer (Coulter Electronics) after conjugation with fluorescein-labelled monoclonal antibodies CD2 (mature T-cells), CD4 (helper/inducer), CD8 (cytotoxic/suppressor), CD25 (IL-2 receptor), CD14 and CD16 (natural killer cells), and HLA-DR. Target cells for the assays of lymphocyte natural killer cytotoxic activity (NK) were the human erythroleukemia cell line K562 (a natural killer-sensitive cell line) and for the lymphokine activated killer cell activity (LAK) the Burkitt's lymphoma cell line Daudi (a natural killer-resistant cell line). Target cells were labelled by adding 100 $\mu$Ci $^{51}Cr/10^7$ cells and incubating for 1 h at 37°C. The cells were washed twice and then incubated for 30 min at 37°C in RPMI culture medium. Following this incubation, the labelled cells were washed two more times and $2 \times 10^5$ cells in 50 $\mu$l of medium were added to the wells of a round bottom tissue culture microplate. Effector cells were added to make final effector:target cell ratios of 40:1, 10:1, 2.5:1. Plates were briefly centrifuged and then incubated at 37°C for 4 h. Supernatants were removed using a Skatron harvesting apparatus (Skatron, Lier, Norway) and the radioactivity was determined with a gamma counter. A solution of 0.1 N HCl and culture medium were used instead of effector cells to determine maximal (MR) and spontaneous (SR) release, respectively, of radioactivity from target cells. The percentage of specific tumour cell lysis was calculated using the formula:

$$\%\ \text{Specific lysis} = \frac{\text{c.p.m. sample} - \text{c.p.m. SR}}{\text{c.p.m. MR} - \text{c.p.m. SR}} \times 100$$

All assays were done in quadruplicate.

Statistical significance was assessed with two-group $t$ test and the paired two sided $t$-test.

An increase of NK and LAK activities over pretreatment levels was seen after the first 2 weeks of treatment. This increase did not reach statistical significance (Figure 1). After the 6-week cycle, the mean increase was about 50% for NK activity ($P<0.005$) and 345% for LAK activity ($P<0.01$), respectively. The behaviour of individual patients is illustrated in Figure 2. Enhanced NK activity was seen in ten of 12 patients and enhanced LAK activity in 11 of 12 patients, unrelated to the dose of IL-2 administered. The number of total lymphocytes and the percentages of lymphocytes subsets after 6 weeks of treatment did not show variations with respect to baseline values (not shown).

Toxicity was limited to WHO grade I and II except for one patient who developed grade III neurological toxicity (somnolence lasting >50% of waking hours) regressing within 2 days from the suspension of treatment. More frequent side effects were fever, chills, malaise and fatigue. The subcutaneous administration of IL-2 resulted in transient

Correspondence: S. Iacobelli, Cattedra Oncologia Medica, Universita' 'G. D'Annunzio', Via dei Vestini 66, 66100 Chieti, Italy.
Received 23 December 1991; and in revised form 1 May 1992.

**Table I** Patient characteristics

| *Pts No.* | *Age (yrs)* | *Sex* | *Performance status* | *Prior treatment* | *Diagnosis* | *Dose if IL-2 IU m² die* |
|---|---|---|---|---|---|---|
| 1 | 25 | M | 1 | CT + IT | Cutaneous melanoma | 240,000 |
| 2 | 62 | M | 2 | RT + CT | Small cell lung carcinoma | 240,000 |
| 3 | 65 | F | 0 | IT | Ocular melanoma | 240,000 |
| 4 | 39 | F | 0 | CT | Ocular melanoma | 240,000 |
| 5 | 60 | M | 1 | None | Ocular melanoma | 600,000 |
| 6 | 48 | F | 1 | CT | Fallopian tubes carcinoma | 600,000 |
| 7 | 61 | M | 1 | None | Kidney carcinoma | 1,200,000 |
| 8 | 70 | M | 1 | CT | Pancreas carcinoma | 1,200,000 |
| 9 | 43 | M | 1 | CT + IT | Kidney carcinoma | 1,200,000 |
| 10 | 57 | M | 1 | CT | Hepatocarcinoma | 1,200,000 |
| 11 | 60 | F | 1 | None | Kidney carcinoma | 2,400,000 |
| 12 | 57 | M | 0 | CT + IT | Kidney carcinoma | 2,400,000 |

CT, chemotherapy; IT, immunotherapy; RT, radiotherapy.

**Figure 1** Mean levels of NKI **a**, and LAK **b**, activity in all patient population evaluated before treatment (—●—), after 2 weeks (—■—), and at the end (—▲—) of a treatment at various Effector:Target ratios (see 'Materials and methods'). *P* values *vs* baseline by paired *t* test: * <0.05; ** <0.01; *** <0.005; ns, not significant.

**Figure 2** Levels of NK **a**, and LAK **b**, activity in individual patients before (■) and at the end (□) of the 6-week treatment cycle at a fixed Effector:Target ratio of 40:1. *P* values *vs* baseline (two-group *t*-test): * < 0.02; ** <0.002; ns, not significant.

inflammation and local induration at the injection site, which persisted for up to 2 weeks after treatment. However, none of the patients judged this side-effect as unacceptable. No ulceration occurred.

Three patients with renal-cell carcinoma and one with ocular melanoma showed disease stabilisation for at least 3 months. No objective responses were observed.

Our combination regimen containing low IL-2 doses was able to induce immunological changes. Significant enhancement of NK and LAK activity was observed in the majority of the patients. The extent of the immune activation was about of the same order of magnitude as that observed using much higher IL-2 doses (Rosenberg, 1986; Sondel, 1988). This further supports the concept previously expressed by others (Hank *et al.*, 1988; Rosenthal *et al.*, 1988; Urba *et al.*, 1990) that long term chronic exposure to low dose IL-2 might be more efficient than short-term stimulation by high-dose to obtaining significant immunological effects.

The stimulation of immune functions was already evident at the lowest IL-2 doses. Four patients received more than one course of therapy. Among these, two patients enrolled at the beginning of the study receiving 240,000 and 600,000 IU m² day, respectively, showed maximal stimulation of LAK activity that persisted during three consecutive 6-week cycles (not shown). Patient heterogeneity with regard to tumour burden, tumour type, and previous treatment may explain why enhanced cellular cytotoxicity was seen in some patients but not in others independently of the amount of IL-2 administered. One additional cause of heterogeneity could be the variable production of neutralising antibodies on IL-2 activity *in vivo* as evidenced in a recent report (Whitehead *et al.*, 1990). These same reasons could explain our inability to consistently see alterations in the distribution of lymphocyte subsets.

In summary, this investigation shows that long-term sequential weekly IFN-a and low-dose IL-2 possesses immunological activity in patients with cancer. The regimen is provided with low toxicity, and prolonged treatment is possible with little inconvenience to the patient. Since patients can be easily trained to give their own s.c. IL-2 injections (Atzpodien *et al.*, 1990*b*; Stein *et al.*, 1991), our regimen may be suitable for study as possible adjuvant in the treatment of IL-2 sensitive malignancies.

### References

ATZPODIEN, J. & KIRCHNER, H. (1990*a*). Cancer, cytokines and cytotoxic cells: interleukin-2 in the immunotherapy of human neoplasms. *Klin Wochenschr.*, **61**, 1-11.

ATZPODIEN, J., KORFER, A., FRANKS, C.R., POLIWODA, H. & KIRCHNER, H. (1990*b*). Home therapy with recombinant interleukin-2 and interferon-alpha2b in advanced human malignancies. *Lancet*, **335**, 1509-1512.

CAMERON, R.B., MCINTOSH, J.K. & ROSENBERG, S.A. (1988). Synergistic antitumor effects of recombinant immunotherapy with interleukin-2 and a recombinant hybrid alpha-interferon in the treatment of established murine hepatic metastases. *Cancer Res.*, **48**, 5810-5817.

FALTYNEK, C.R. & OPPENHEIM, J.J. (1988). Interferons in the Host Defence. *J. Natl Cancer Inst.*, **80**, 151-153.

FOON, K.A. (1989). Biological response modifiers. The new immunotherapy. *Cancer Res.*, **49**, 1621-1639.

GOLDSTEIN, D., SOSMAN, J.A., HANK, J.A. & 8 others (1989). Repetitive weekly cycles of interleukin-2 on non-major histocompatibility complex-restricted killer activity. *Cancer Res.*, **49**, 6832-6839.

HANK, J.A., KHOLER, P.C., WEIL-HILLMAN, G. & 7 others (1988). *In vivo* induction of the lymphokine-activated killer phenomenon: interleukin2-dependent human non-major histocompatibility complex-restricted cytotoxicity generated *in vivo* during administration of human recombinant Interleukin 2. *Cancer Res.*, **48**, 1965-1971.

IIGO, M., SAKURAI, M., TAMURA, T., SAIJO, N. & HOSHI, A. (1988). *In vivo* antitumor activity of multiple injection of recombinant interleukin-2, alone and in combination with three different types of recombinant interferon on various syngenic murine tumors. *Cancer Res.*, **48**, 260-264.

LEE, R.E., LOTZE, M.T., SKIBBER, J.M. & 7 others (1989). Cardiorespiratory effects of immunotherapy with interleukin-2. *J. Clin. Oncol.*, **7**, 7-20.

LEE, K.H., TALPAZ, M., ROTHBERG, J.M. & 6 others (1989). Concomitant administration of recombinant human interleukin-2 and recombinant interferon alpha-2a in cancer patients: a phase II study. *J. Clin. Oncol.*, **7**, 1726-1732.

PICHERT, G., JOST, L.M., FIERZ, W. & STAHEL, R.A. (1991). Clinical and immune modulatory effects of alternative weekly interleukin-2 and interferon alpha-2a in patients with advanced renal-cell carcinoma and melanoma. *Br. J. Cancer*, **63**, 287-292.

ROSENBERG, S.A. (1986). Adoptive immunotherapy of cancer using lymphokine activated killer cells and recombinant interleukin-2. In De Vita, V.T., Hellman, S. & Rosenberg, S.A. (eds). p. 55-91. *Important Advances in Oncology*. P.A. Lippincott: Philadelphia.

ROSENBERG, S.A., LOTZE, M.T. & MULE', J.J. (1988*a*). New approaches to the immunotherapy of cancer using interleukin-2. *Ann. Intern. Med.*, **108**, 853-864.

ROSENBERG, S.A., SCWARTZ, S. & SPIESS, P.T. (1988*b*). Combination immuno-therapy for cancer. Synergistic antitumor interactions of interleukin-2, alpha-interferon and tumour infiltrating lymphocytes. *J. Natl Cancer Inst.*, **80**, 1393-1397.

ROSENBERG, S.A., LOTZE, M.T., YANG, J.C. & 7 others (1989). Combination therapy with interleukin-2 and alpha-interferon for the treatment of patients with advanced cancer. *J. Clin. Oncol.*, **7**, 1863-1874.

ROSENTHAL, N.S., HANK, J.A., KOHLER, P.C. & 6 others (1988). The *in vitro* function of lymphocytes from 25 cancer patients receiving four to seven consecutive days of recombinant IL2. *J. Biol. Response Modif.*, **7**, 123-129.

SONDEL, P.M., KOHLER, J.A., HANK, J.A. & 5 others (1988). Clinical and immunological effects of recombinant interleukin-2 given by repetitive weekly cycles to patients with cancer. *Cancer Res.*, **48**, 2561-2567.

STEIN, R.C., MALKOVSKA, V., MORGAN, S. & 8 others (1991). The clinical effects of prolonged treatment of patients with advanced cancer with low-dose subcutaneous interleukin 2. *Br. J. Cancer.*, **63**, 275-278.

URBA, W.J., STEIS, R.G., LONGO, D.L. & 6 others (1990). Immunomodulatory properties and toxicity of Interleukin 2 in patients with cancer. *Cancer Res.*, **50**, 185-192.

WHITEHEAD, R.P., WARD, D., HEMINGWAY, L., HEMSTREET III G.P., BRADLEY, E, & KONRAD, M. (1990). Subcutaneous recombinant interleukin 2 in a dose escalating regimen in patients with metastatic renal cell adenocarcinoma. *Cancer Res.*, **50**, 6708-6715.

WHO GENEVA (1979). WHO Handbook for reporting results of cancer treatment WHO Offset Publication No. 48, WHO: Geneva.